# Probing the Molecular Mechanism of Viscoelastic Relaxation in Transient Networks

**DOI:** 10.3390/gels9120945

**Published:** 2023-12-01

**Authors:** Shota Michida, Ung-il Chung, Takuya Katashima

**Affiliations:** 1 Department of Material Engineering, Faculty of Engineering, The University of Tokyo, 7-3-1 Hongo, Bunkyo-ku, Tokyo 113-8656, Japan; michida-shota1681@g.ecc.u-tokyo.ac.jp; 2 Department of Bioengineering, School of Engineering, The University of Tokyo, 7-3-1 Hongo, Bunkyo-ku, Tokyo 113-8656, Japan; tei@tetrapod.t.u-tokyo.ac.jp; 3 Center for Disease Biology and Integrative Medicine, Graduate School of Medicine, The University of Tokyo, 7-3-1 Hongo, Bunkyo-ku, Tokyo 113-8656, Japan

**Keywords:** associative polymer, transient network, viscoelasticity, fluorescence recovery after photobleaching, surface plasmon resonance

## Abstract

Hydrogels, which have polymer networks through supramolecular and reversible interactions, exhibit various mechanical responsibilities to its surroundings. The influence of the reversible bonds on a hydrogel’s macroscopic properties, such as viscoelasticity and dynamics, is not fully understood, preventing further innovative material development. To understand the relationships between the mechanical properties and molecular structures, it is required to clarify the molecular understanding of the networks solely crosslinked by reversible interactions, termed “transient networks”. This review introduces our recent progress on the studies on the molecular mechanism of viscoelasticity in transient networks using multiple methods and model materials. Based on the combination of the viscoelasticity and diffusion measurements, the viscoelastic relaxation of transient networks does not undergo the diffusion of polymers, which is not explained by the framework of conventional molecular models for the viscoelasticity of polymers. Then, we show the results of the comparison between the viscoelastic relaxation and binding dynamics of reversible bonds. Viscoelastic relaxation is primarily affected by “dissociation dynamics of the bonds” and “network structures”. These results are explained in the framework that the backbone, which is composed of essential chains supporting the stress, is broken by multiple dissociation events. This understanding of molecular dynamics in viscoelasticity will provide the foundation for designing transient networks.

## 1. Introduction

Recently, numerous studies have delved into the design of responsive hydrogels incorporating non-covalent bonds, particularly supramolecular and reversible interactions. These responsive hydrogels are essential in creating self-healing and robust soft materials, garnering significant attention [1,2,3,4]. Reversible crosslinks, such as coordinate bonds, hydrogen bonds, and dynamic covalent bonds, continuously bind and dissociate, giving hydrogels dynamic properties like viscoelasticity. Understanding the effects of these reversible bonds on the viscoelasticity and molecular dynamics of hydrogels is generally complex and remains incomplete. The incomplete understanding prevents further innovative materials development. It is crucial to clarify the relationships between the mechanical properties and the molecular structures of polymer networks crosslinked by reversible bonds, commonly referred to as “transient networks”. These networks are three-dimensional structures formed with reversible crosslinks that have a finite lifetime. Because of their unique composition, transient networks show the elasticity in the short time range, while it can flow in the long time range, classifying them as viscoelastic “liquids” from the viewpoint of rheology.

There have been many theoretical and experimental attempts to elucidate the relationships between the viscoelasticity and molecular structures in transient networks. In 1946, Green and Tobolsky introduced a “phenomenological model (GT model)” for the viscoelastic liquids, where the elasticity originated from the network structure formed through the reversible crosslinks [5]. In the GT model, because the crosslinks have a finite lifetime (*τ*_d_), the system shows the elasticity of polymer gels and rubbers against a faster deformation than the lifetime. On the other hand, it exhibits the fluidity against a slower deformation than the lifetime. The remarkable feature of the GT model is that it interprets viscoelastic liquids not as extensions of viscous bodies but rather as extensions of a network of elastic bodies. However, the lifetime was introduced phenomenologically without any molecular picture. Thus, the relationships between the viscoelasticity and molecular structures, such as the non-Newtonian behaviors, including shear thickening and thinning [6,7], remain ambiguous. After his works, there have been many attempts to address and explain the relationships between the viscoelasticity and molecular dynamics based on the GT model. For example, Tanaka and Edwards reported that the lifetime is determined by the balance between the bond dissociation and recombination. While these modifications allowed for the calculation of the complex rheological properties of transient networks, the molecular interpretation of viscoelasticity remained obscure, owing to the existence of some fitting parameters in these models [8,9,10,11,12,13,14].

On the other hand, the “molecular models” of polymeric liquid have been developed in the rubbery state, including entangled polymer melts and solutions. In these materials, the deviatoric parts of the stress tensor and optical anisotropic tensor are proportional [15,16], which is well known as the stress-optical rule (SOR). In soft material, the SOR allows us to relate the elasticity of the polymeric liquid stems from the orientational anisotropy of polymer chains with entropic elasticity between the ends. Meanwhile, viscoelastic relaxation is predominantly governed by the disappearance of the anisotropy, facilitated by the Brownian motion of each chain. Here, we focus on the isotropic materials, where the Brownian motion of the polymers through the thermal fluctuation is allowed. Consequently, the dynamics of polymers can be approximated by the random walk process of diffusion. Therefore, according to the Langevin equation, the time required for the clearance (*τ*) is represented as [17,18]:

(1) $$\tau =\frac{R^{2}}{D_{rotational}}$$

where *R* is the average end-to-end distance of the polymer, and *D*_rotational_ is the rotational diffusion coefficient. Rotational diffusion is the rotational motion that acts on polymers present in a fluid, resulting from random changes in their orientation. As a concept opposite to rotational diffusion, there is translational diffusion, which is the motion of the center of the gravity. The translational diffusion coefficient, *D*_translational_, has a correlation with *D*_rotational_. Thus, Equation (1) implies that the viscoelastic relaxation time follows the time polymers take to translationally diffuse to its self-size. This has been widely verified in entangled polymer systems and certain particle suspensions [18,19].

However, in the field of transient networks, many researchers believe that the lifetime of bonds agrees with the dissociation time of bonds [20,21]. There have been few reports to investigate the relationships between the viscoelasticity and the diffusion dynamics. As a result, the molecular understanding of viscoelasticity in transient networks remains incomplete. 

Up until now, our groups have focused on elucidating the molecular understanding of the viscoelasticity in transient networks based on multiple methods [22,23]. In this review, we introduce the recent progress on this field using two model transient networks: hydrophobically ethoxylated modified urethane (HEUR) and Tetra-PEG slime. The former is one of the associative polymers, which has been utilized by many researchers [24,25,26,27,28,29]. The latter is a class of transient networks with regular network structures developed by our group, comprising of the multi-armed prepolymers [30]. Our findings afford a deeper understanding of the molecular mechanisms involved in the relaxation of transient networks, leading to a more refined material design.

## 2. Relationships between Diffusion and Viscoelasticity of Associative Polymer Networks

HEUR consists of a hydrophilic main chain and hydrophobic end groups. When in water, the end groups aggregate via hydrophobic interactions, enabling the formation of transient networks above a certain concentration. HEUR networks are well known to exhibit viscoelastic relaxation consistent with the Maxwellian model with a single relaxation mode [24,25,26,31,32,33,34,35]. Owing to their mechanical simplicity, HEUR networks have been utilized as model systems for transient networks.

Here, to compare the viscoelastic relaxation time with the time for diffusion in Equation (1), we performed the dynamic viscoelastic measurements and the fluorescence recovery after photobleaching (FRAP), respectively. For the evaluation of the diffusion coefficient, three main methods have been reported. One is the dynamic light-scattering (DLS) technique, where the fluctuation of scattering light from the polymer is detected. Because the autocorrelation function obtained from the fluctuation data is derived from the Brownian motion of the polymer, the diffusion coefficient can be estimated based on the relaxation time of the autocorrelation function [36,37,38,39,40,41,42,43]. The advantage of the DLS is that it is invasive and easier to measure using light scattering than other methods. However, the “translational diffusion” of the polymer can be evaluated by DLS only for the diluted polymer solution, where the polymers are isolated in the system. It is not adequate to measure the translational diffusion in concentrated solutions or networks because the cooperative diffusive mode is pronounced [44,45,46,47,48]. Another one is the pulsed-field gradient nuclear magnetic resonance (PFG-NMR), which utilizes magnetic gradient fields to induce phase differences in nuclear spins, providing positional information for protons in a specific direction. Following the removal of the gradient field, echo signals decay due to self-diffusion. In practical measurements, signal intensity and pulse intervals are adjusted. The obtained relationships between these parameters are used to evaluate the diffusion coefficient [49,50,51,52,53,54,55,56]. PFG-NMR is advantageous, as it detects proton diffusion without requiring chemical modification. However, it may be less effective in measuring slow diffusion, especially within transient networks, as diffusion times slower than the *T*_2_ relaxation time are challenging to observe. The other one is fluorescence recovery after photobleaching (FRAP), involving the chemical modification of molecules with fluorescent probes. These molecules, located within a limited area, are exposed to high-intensity laser beams, leading to photobleaching. The self-diffusion coefficient is evaluated from the time-dependent recovery of intensity due to molecular self-diffusion. Although one should examine the effect of modification, the FRAP can detect the wide-ranged diffusion coefficient [57,58,59,60,61].

**Figure 1:**
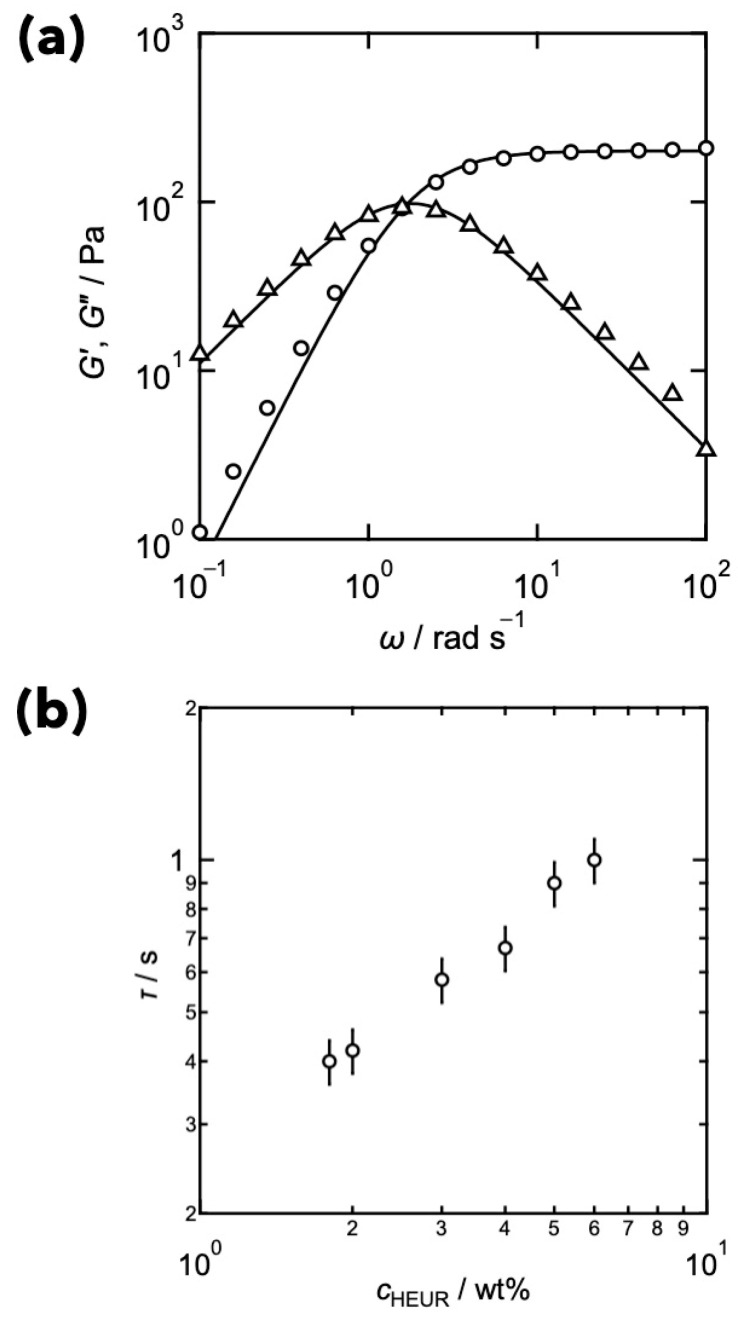
(**a**) Representative data of angular frequency dependence of storage (circles) and loss (triangles) moduli of the HEUR at 25 °C. The solid lines represent the prediction of the Maxwellian model. (**b**) Viscoelastic relaxation time as a function of HEUR concentration (*c*_HEUR_). (Reproduced from Ref. [63] with permission).

Figure 1a shows the representative results of the dynamic viscoelasticity. The storage (*G*′) and loss (*G*″) moduli are displayed as circles and triangles, respectively. In high frequencies, *G*′ was higher than *G*″, while they showed the crossover at around *ω* ≈ 10^0^ rad s^−1^, suggesting that it was viscoelastic liquid. The solid lines represent the prediction of the Maxwellian model, shown as [62]:

(2a) $$G^{\prime }=\Delta G\frac{\omega^{2}\tau^{2}}{1+\omega^{2}\tau^{2}}$$

(2b) $$G^{\prime \prime }=\Delta G\frac{\omega \tau }{1+\omega^{2}\tau^{2}}$$

where Δ*G* is the relaxation strength, and *τ* is the viscoelastic relaxation time. The solid lines agree with the experimental results, indicating that the viscoelastic relaxation of the HEUR underwent a unique process. Figure 1b shows the estimated relaxation time as a function of HEUR concentration. The viscoelastic relaxation time increased with the increase in the concentration.

**Figure 2:**
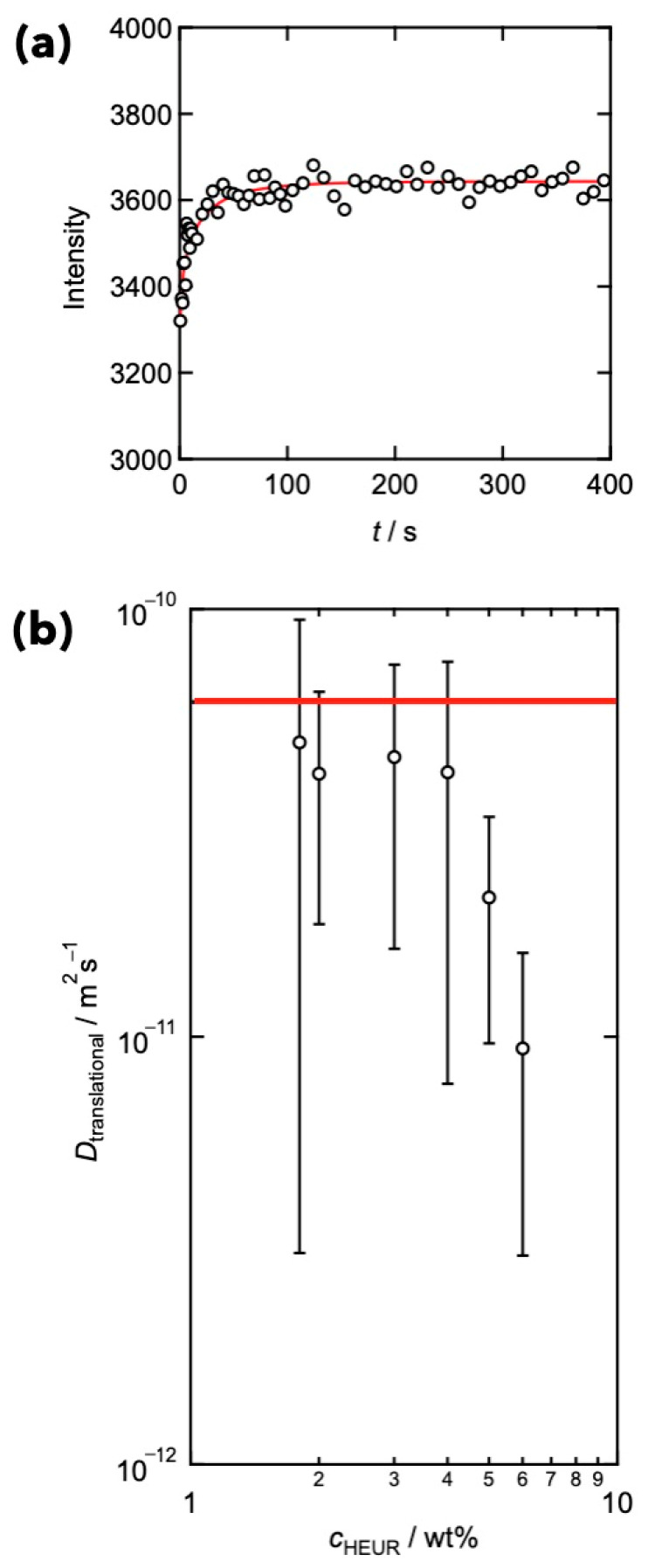
(**a**) Representative FRAP data for HEUR. The red line represents the fitting line by Equation (3). (**b**) Estimated translational diffusion coefficient (*D*_translational_) as a function of HEUR concentration (*c*_HEUR_). (Reproduced from Ref. [63] with permission).

Figure 2a shows the representative data of FRAP, where the time development of the intensity of the beaching area was large enough to evaluate the translational diffusion. After the bleaching, the intensity recovered gradually and approached the original value at 300 s. Here, we fit the data using the stretched exponential function (Kohlrausch–Williams–Watts (KWW)-type equation) as

(3) $$F=A-Bexp\left[-{\left(\frac{t}{\gamma \tau_{KWW}}\right)}^{\beta }\right]$$

where *A* and *B* are the original intensity and the reduction in intensity, *τ*_KWW_ is the characteristic time for the recovery, *β* is the exponent indicating the polydispersity of *τ*_KWW_, and *γ* (=1.5) accounts for nonidealities of the spatial heterogeneity in laser intensity. (The details are described in our previous paper [63]). Based on the values of *τ*_KWW_, the translational diffusion coefficient (*D*_translational_) was calculated using:

(4a) $$D_{translational}=\frac{d^{2}}{4\langle \tau_{w}\rangle }$$

(4b) τw=Γ2βΓ1βτKWW

Here, d is the bleaching area (diameter of 100 µm), and <*τ*_w_> is the second-order average of *τ*_KWW_. It should be noted that the values of *D*_translational_ were confirmed to be independent of *d*, indicating that the translational diffusion was dominant. Figure 2b depicts the relationships between *D*_translational_ and polymer concentration in HEUR. The solid line signifies the data for HEUR in a methanol solution, where hydrophobic interactions were screened. The obtained *D*_translational_ was lower, compared to that in methanol, implying that the associations limited chain mobility.

**Figure 3:**
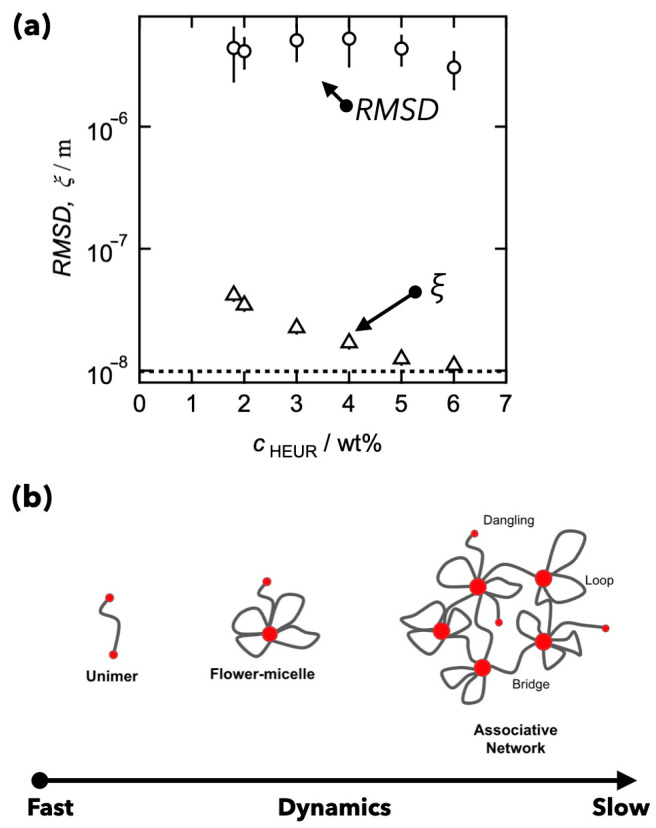
(**a**) Relationships between RMSD (circles) and *ξ* (triangles) as a function of HEUR concentration. (**b**) Schematic illustration of dynamic heterogeneity in transient networks. (Reproduced from Ref. [63] with permission).

Figure 3a displays the root-mean-square distance (*RMSD*) of polymers during viscoelastic relaxation time. Under the assumption of a random walk, *RMSD* is derived as:

(5) $$RMSD={\left(D_{translational}\tau \right)}^{1/2}$$

The random walk assumes that that the polymer chain moves in isotropic, independent movements in a three-dimensional space, with equal probabilities for each step, no memory of previous configurations, and excluded volume effects neglected. *RMSD* is independent of concentration and 100 times larger than the precursor chain size represented as the dashed line (~10^−8^ m). Part of this deviation is attributed to the superbridge structure, where HEUR networks form by connecting flower-like micelles. This results in multiple loop structures with effective network strands composed of numerous strands. Here, the effective network strand length, *ξ*, is approximated using the affine network model [64] as:

(6) $$\xi =\frac{k_{B}T}{G}$$

where *k*_B_, *T*, and *G* are the Boltzmann constant, the absolute temperature, and the shear modulus, respectively. As depicted, *RMSD* remains 10–100 times longer than the distance between the crosslinks, *ξ*. There are two possible reasons for the discrepancies between *RMSD* and *ξ*: one is the breaking of the concept of Equation (2), and the other is attributed to the “dynamic heterogeneity” in transient networks. Dynamic heterogeneity is defined as the spatiotemporal fluctuations in local dynamical behavior, which are often observed in glass-forming materials [65,66,67]. HEUR networks comprise not only superbridge structures but also unimers and flower micelles, contributing to dynamic heterogeneity (refer to Figure 2). The diffusion process is principally governed by components that diffuse quickly. In areas of low concentration, unimers and flower micelles are predominant, facilitating rapid diffusion. Conversely, the network component, contributing to viscoelasticity, diffuses at a slower rate. In areas with high concentration, molecules of HEUR could potentially diffuse more swiftly than the network component, due to the recombination of the HEUR chains’ aggregation cores.

The studies involving HEUR have demonstrated that the viscoelastic relaxation time is not explained in the conventional framework, based on the diffusion of polymers expressed as Equation (2). The reason for the deviation remains ambiguous, due to the inherent structural heterogeneity of the experimental systems. 

## 3. Relationships between Translational Diffusion and Viscoelasticity in Transient Networks with Controlled Network Structures

**Figure 4:**
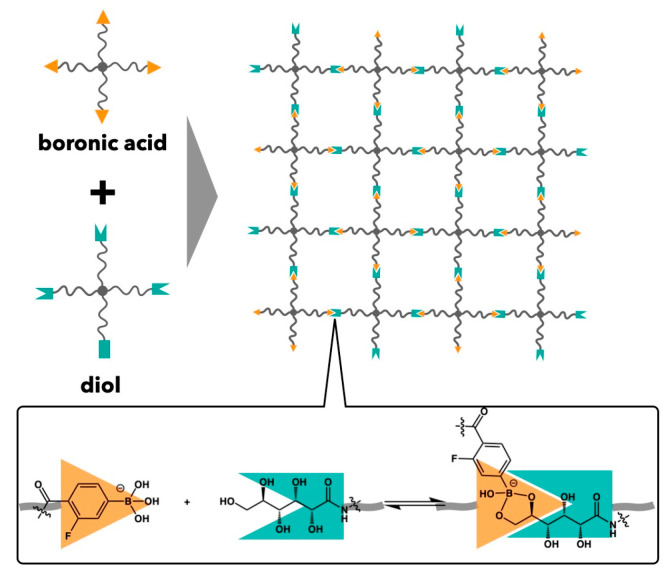
Schematic illustration of Tetra-PEG slime, which is formed through the reversible reaction between boronic acid and diol end groups.

Based on the prior section, there is a clear necessity for model materials with precisely controlled network structures to elucidate molecular mechanisms. Recently, we developed model transient networks utilizing tetrafunctional precursors (Tetra-PEG slimes) by employing dynamic covalent bonds between 4-carboxy-3-fluorophenylboronic acid (FPBA) and glucono-δ-lactone (GDL) as reversible crosslinks [30,68,69,70]. Due to the temporal characteristics of the crosslinkers, the Tetra-PEG slime exhibits the flow behavior in a long time limit, classified into transient networks. Tetra-PEG slime is formed by mixing two types of star-shaped precursor aqueous solutions (*M*_w_ = 10, 20 kg mol^−1^, concentration = 40–160 g L^−1^). The precursors have a narrow distribution of mass, facilitating the formation of networks with static homogeneity, such as regular structures, uniform functionality, and strand length. Additionally, the employment of symmetric precursors possessing uniform diffusibility is attributed to dynamic homogeneity. These static and dynamic homogeneities, which were unavailable in HEUR models, minimize the variability of structural parameters (refer to Figure 4). The details of preparation conditions were described in our previous studies [30].

**Figure 5:**
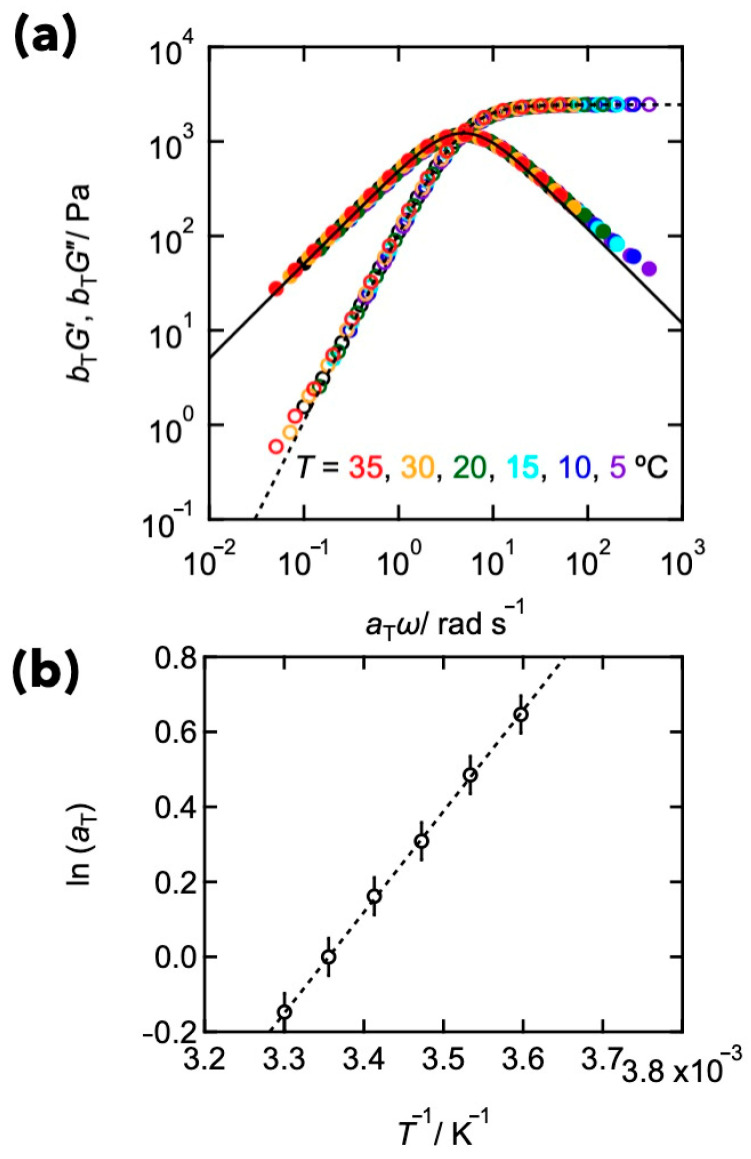
(**a**) Composite curves showing the frequency dependence of the storage and loss moduli for the dynamically cross-linked Tetra PEG gel (*C*_PEG_ = 80 g L^−1^, *M*_pre_ = 10,000 g mol^−1^, and pH 7.4). The measured temperatures were in the range of 5–35 °C. The reference temperature was 25 °C. The solid and dashed lines represent the fit results of the Maxwellian model. (**b**) Time–temperature shift factors *a*_T_ for the function of *T*^−1^. A dashed line represents the fitting line of the Arrhenius equation. (Reproduced from Ref. [30] with permission).

Here, we utilized the Tetra-PEG slime as a model system and investigated the relationships between the viscoelastic relaxation and the diffusion of polymers, following the previous section. As for the viscoelasticity, Figure 5a shows composite curves of the storage and loss moduli (*G*′ and *G*″, respectively) of the Tetra-PEG slime (polymer concentration: 80 g L^−1^, molar mass of precursor: 10,000 g mol^−1^, and pH 7.4) at a reference temperature of 20 °C. The composite curves were obtained by shifting *G*′ and *G*″ data at various temperatures, horizontally and vertically, to superpose the low-frequency data. *a*_T_ and *b*_T_ represent the horizontal and vertical shift factors, respectively. Time–temperature superposition (tTS) fitted well, and the composite curves agreed well with the prediction of Maxwellian models like the HEUR. The suitability of tTS demonstrates that temperature uniformly accelerates all molecular dynamics without any structural alteration within the observed temperature range. Figure 5b shows that the natural logarithm of the horizontal shift factor (ln *a*_T_) was potted against *T*^−1^. On the semilogarithmic plot, ln *a*_T_ increased linearly with *T*^−1^, suggesting that the viscoelastic relaxation follows the Arrhenius behavior as

(7) $$a_{T}=A\;\exp\left(-\frac{E_{a}}{RT}\right)$$

where *E*_a_ is the activation energy. The activation energy was estimated to be 45 kJ mol^−1^.

**Figure 6:**
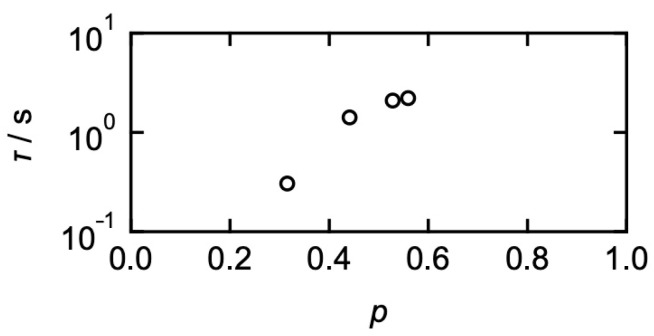
Connectivity (*p*) dependence of the viscoelastic relaxation time (*τ*). (Reproduced from Ref. [68] with permission).

In this study, we tuned the network connectivity using imbalanced mixing of the precursor chains. Figure 6 shows the viscoelastic relaxation time (*τ*) as a function of network connectivity (*p*). *p* is defined as the ratio of the connected end groups of prepolymers against the total end groups at the equilibrium state. *p* is estimated as [68,70]:

(8) $$p=\left\{1+\frac{1}{C_{end}K}\right\}-{\left[{\left\{1+\frac{1}{C_{end}K}\right\}}^{2}-4s\left(1-s\right)\right]}^{1/2}$$

where *C*_end_ and *K* are the total end group concentration and the equilibrium constant between FPBA and GDL, respectively. *s* represents the mixing fraction of two precursors.

**Figure 7:**
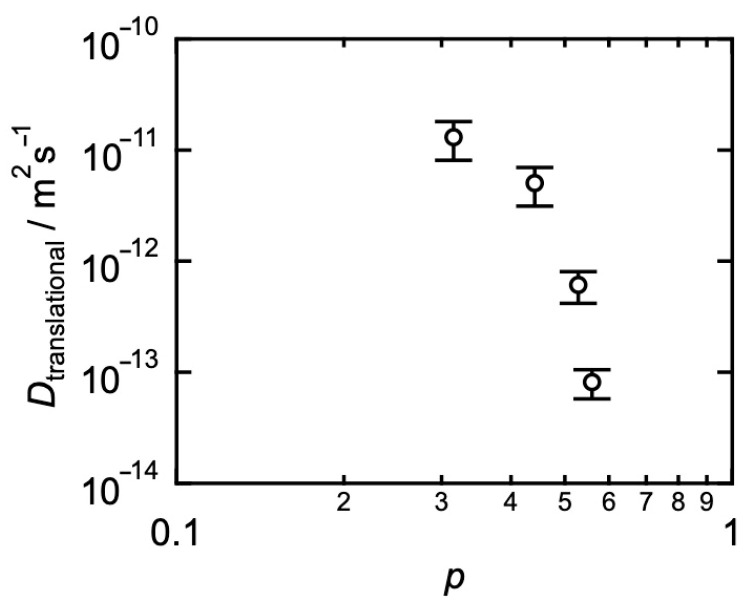
*p*-dependence of the estimated translational diffusion coefficient (*D*_translational_). (Reproduced from Ref. [68] with permission).

As for the diffusion, in Figure 7, the estimated *D*_translational_ was plotted against *p*. Here, the bleaching diameter was 80 μm, where *D* was constant and almost independent of the diameter. Using Equations (4) and (5), the translational diffusion coefficient (*D*_translational_) was determined. *D*_translational_ decreased with increasing *p*, indicating that the collision between stickers restricts the dynamics of polymers.

**Figure 8:**
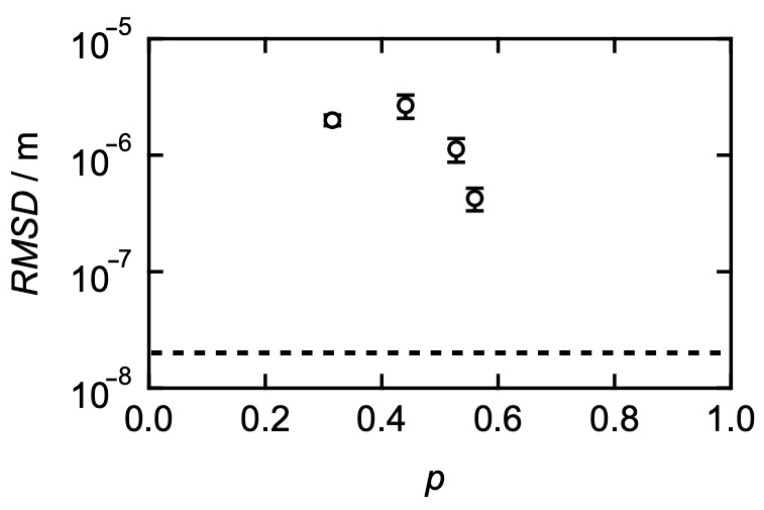
*p*-dependence of root-mean-square distance the prepolymers diffuse during the viscoelastic relaxation time (*RMSD*). The dashed line represents the gyration radius of a four-armed precursor chain. (Reproduced from Ref. [68] with permission).

To directly compare both the viscoelastic relaxation time and the transitional diffusion coefficient, we discuss the root-mean-square distance the prepolymers diffuse during the viscoelastic relaxation time (*RMSD*) using Equation (5). Figure 8 shows the *p*-dependence of *RMSD. RMSD* is an order of 10^−6^ m, which is 100 times larger than the gyration radius, which is consistent with the results of HEUR. *RMSD* slightly decreased at the high *p*-region, suggesting that Equation (1) works well only in the high *p*-limit. The viscoelastic relaxation in transient networks with small amounts of connectivity defect does not proceed through the diffusive motion of each prepolymer like the normal polymer liquid.

## 4. Comparison between Binding and Dissociation Kinetics of Crosslinks and Viscoelasticity in Transient Networks with Controlled Network Structures

In this section, we focus on examining more local dynamics, specifically the binding and dissociation times of crosslinks. Typically, microscopic kinetics are observed using spectroscopic methods like nuclear magnetic resonance, infrared, and ultraviolet-visible spectroscopy [71,72,73,74]. However, these techniques predominantly focus on interactions involving specific chemical bond structures and are unable to accommodate the variety of reaction paths. To overcome this limitation, we performed surface plasmon resonance (SPR), a technique capable of evaluating the binding kinetics between ligands fixed to the metal surface in microfluidic devices and the analytes introduced to the device [75]. The detection by adsorption at the surface enables SPR to trace the binding and dissociation without the constraint of intricate chemical structures, rendering it more versatile, compared to traditional spectroscopies.

**Figure 9:**
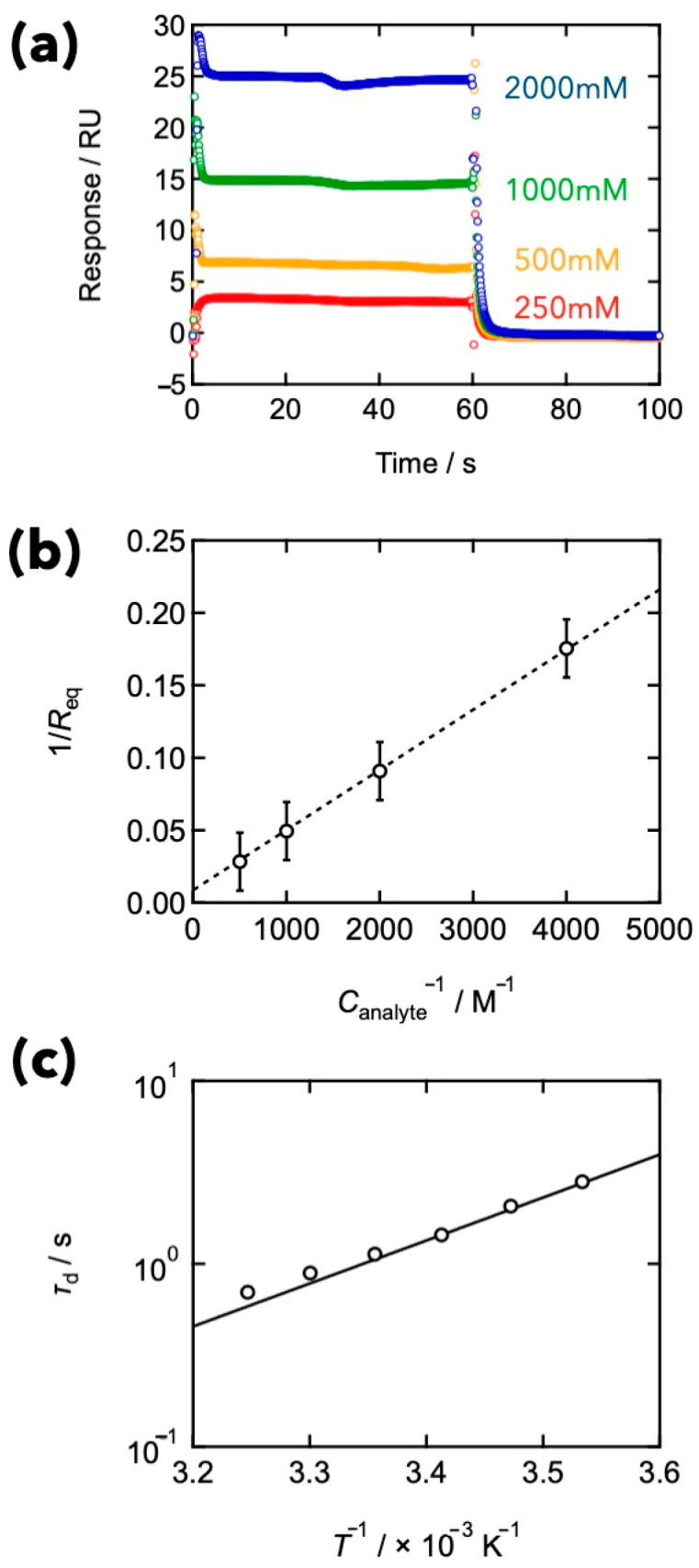
(**a**) Phenylboronic acid-diol binding kinetics with different FPBA concentrations, as assessed by SPR (pH 7.4, and *T* = 25 °C); (**b**) 1/*R*_eq_ against the inverse of the analyte concentration (Benesi–Hildebrand plot). (**c**) Temperature dependence of the estimated dissociation time (*τ*_d_). (Reproduced from Ref. [30] with permission).

Figure 9a illustrates an SPR sensorgram at various analyte (FPBA) concentrations, depicting the kinetics of binding and the dissociation between the FPBA and GDL groups. At *t* = 0–60 s, monofunctional FPBA-modified linear polyethylene glycol (*M*_w_ = 500 g mol^−1^) was introduced as the analyte into the microfluidic device. The SPR signal increased instantly after the injection of the analyte solution and showed the plateau, which suggests that the equilibrium between the binding and dissociation was achieved. At *t* = 60 s, to rinse the system, pure solvent was injected in place of the analyte solution. The signal decreased instantly, reflecting the kinetics of the dissociation.

To evaluate the equilibrium constant *K*, the data at equilibrium state were analyzed. Figure 9b shows the relationship between the inverse of the signal intensity at the equilibrium state (1/*R*_eq_) as a function of the inverse of the analyte concentrations. This plot is known as the Benesi–Hildebrand plot [76]. Assuming one-to-one binding between analyte and ligand molecules, *R*_eq_ can be written as:

(9) $$\frac{1}{R}=\frac{1}{\varepsilon KC_{ligand,0}}\frac{1}{C_{analyte,0}}+\frac{1}{\varepsilon KC_{ligand,0}}$$

where *ε* is the effective contribution to the SPR signal of the complex with unit concentration, and *C*_ligand,0_ and *C*_analyte,0_ are the concentrations of the initial ligand and analyte. Using Equation (10), the value of *K* can be estimated from the slope and intercept of the plot. Here, we estimated *K* to be 208 M^−1^.

From the data of the dissociation process (*t* > 60 s), the dissociation rate constant (*k*_d_) was evaluated by assuming the first-order reaction as

(10) $$R=R_{eq}exp\left(-k_{d}t\right)$$

Figure 9c shows the dissociation time (*τ*_d_), which is the inverse of *k*_d_, at various temperatures. On the semi-logarithmic plot, *τ*_d_ proportionally increased with increasing *T*^−1^, suggesting that the dissociation kinetics followed the Arrhenius equation ((Equation (7)). The activation energy was estimated to be 45 kJ mol^−1^, which is consistent with that of viscoelasticity. From these perspectives, it is fair to conclude that the elementary process of the viscoelasticity is primarily determined by the dissociation. It should be noted that the activation energy in the PEG aqueous solution without the crosslinks was reported to be 15–18 kJ mol^−1^ [77], which is lower than our results. This result also supports the conclusion that the dissociation is dominant for the viscoelasticity of the Tetra-PEG slime.

**Figure 10:**
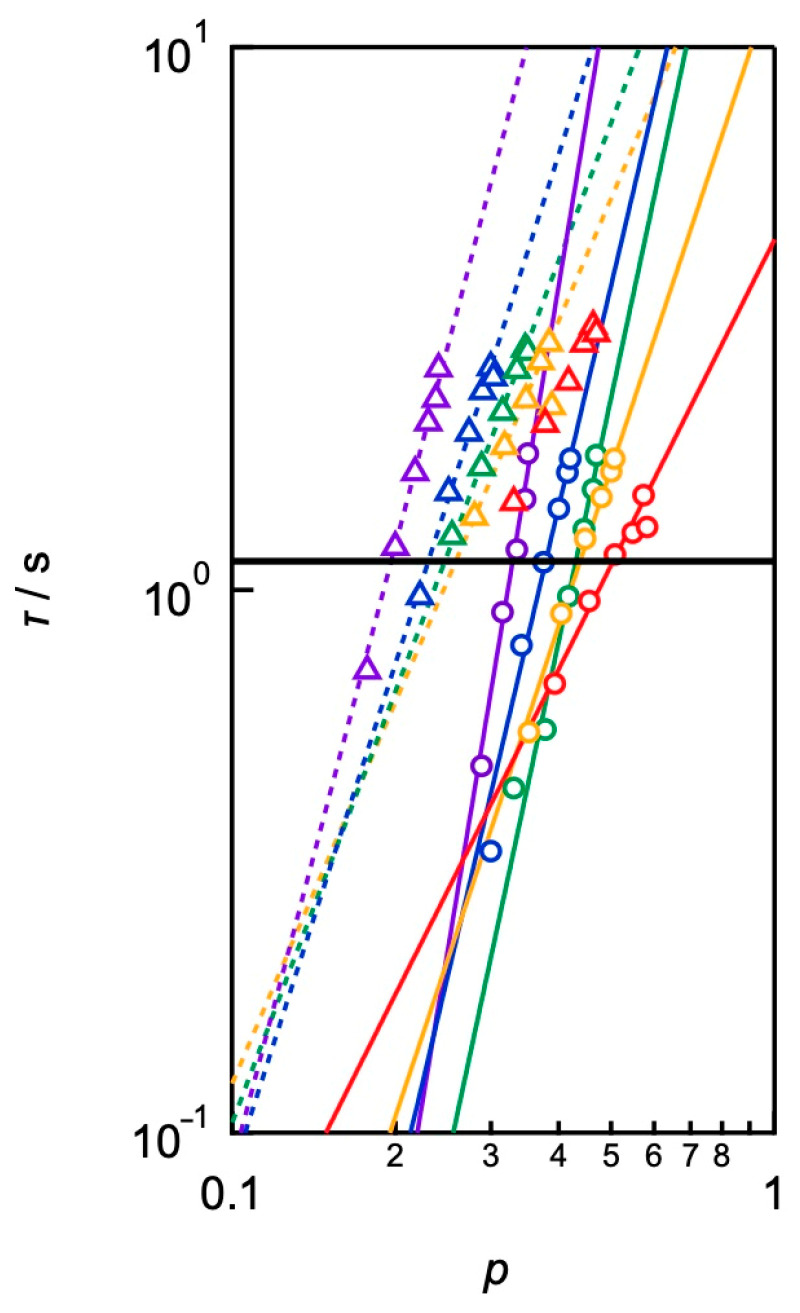
Double logarithmic plot of viscoelastic relaxation time against *p* (circle: *M*_w_ = 10,000 g mol^−1^ and triangle: *M*_w_ =20,000 g mol^−1^). The measured concentrations were in the range of 40–160 g L^−1^ (purple: 40 g L^−1^, blue: 60 g L^−1^, green: 80 g L^−1^, yellow: 100 g L^−1^, and red: 160 g L^−1^). The solid line represents the bond lifetime estimated by SPR (*τ*_d_). (Reproduced from Ref. [70] with permission).

Here, we obtained the viscoelastic relaxation time and dissociation time independently and can directly compare them to discuss the molecular origin. Figure 10 shows the *p*-dependence of *τ* at different concentrations and strand lengths, showing that *τ* increased with increasing *p* for every sample. In conventional transient network systems, *p* is dependent on the polymer concentration and network strand length. Therefore, Figure 10 is the first example of the *p*-dependence of the relaxation time without the effects of the polymer concentration and network strand length. In the figure, the horizontal solid line represents the dissociation time (*τ*_d_) estimated by SPR. In the low *p*-regions, *τ* is faster than *τ*_d_. Additionally, the *p*-value that *τ* matches with *τ*_d_ shifts to higher *p*-regions with increasing polymer concentrations.

**Figure 11:**
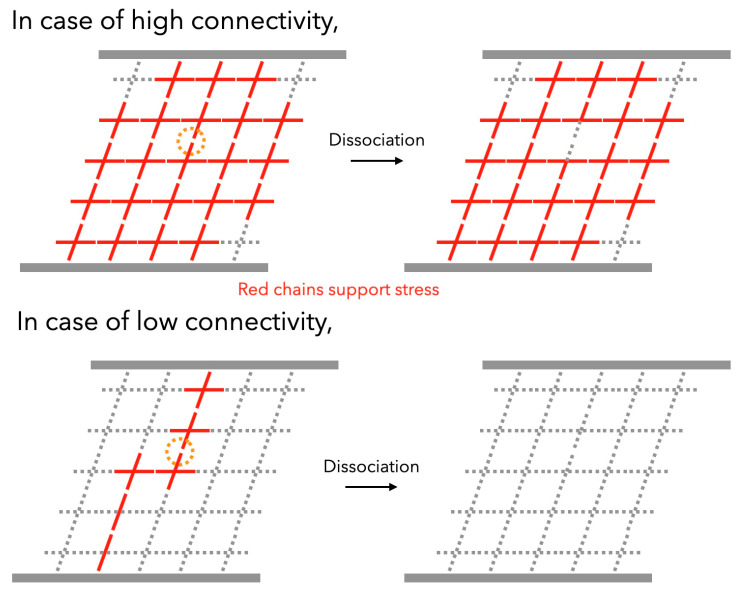
Schematic illustration of viscoelastic relaxation in transient networks through the relaxation of the backbone in case of high connectivity (**top**) and low connectivity (**bottom**). In the high connectivity, almost all the chains support the stress and work as the backbone (represented by red chains), where one dissociation event cannot kill the backbone. On the other hand, the backbone in the low connectivity can be killed by a dissociation event. (Reproduced from Ref. [70] with permission).

The correlation of *τ* to *p* can be interpreted through the concept of the “backbone,” the primary strand bearing stress (see in Figure 11). In a situation of high connectivity, the backbone is comprised of almost all the chains, where one dissociation event cannot kill the backbone due to supplementary bonds. Consequently, the increase in *p* signifies enhanced backbone durability. Conversely, near the gelation point where *p* decreases, the backbone consists of bonds sequentially interconnected by reversible bonds, meaning the backbone disintegrates instantly upon one bond dissociation. According to first-order kinetics, the survival probability [*P*_bond_ (*t*)] of the bond after a specific time (*t*) is expressed as:

(11a) $$P_{bond}\left(t\right)=exp\left(-\frac{t}{\tau_{d}}\right)$$

When the backbone comprises *N*-bonds, it possesses *N*-dissociable points, and the survival probability [*P*_backbone_ (*t*)] of the backbone after a time period can be represented as:

(11b) $$P_{backbone}\left(t\right)=exp\left(-N\frac{t}{\tau_{d}}\right)$$

This implies that the backbone’s lifetime contracts with the accretion in the number of reversible bonds forming the backbone, relative to the dissociation time. It should be noted that the time required for binding is significantly longer than the time required for the self-size diffusion of polymer chains. This means that the effect of chains self-assembling is negligible.

Furthermore, the obtained results where the viscoelastic relaxation time depended on the network connectivity showing the power-law behavior is qualitatively consistent with some theoretical models [78,79]. These models are also based on the concept of the backbone. They predicted the backbone structures using the fractal dimension under the assumption of the random-branching process. It should be noted that the detailed exponents of the power law do not coincide with the predictions. It may be attributed to the potential inaccuracies in the random-branching process in experimental conditions. In low polymer concentrations, such as the dilute and semi-dilute regions where reactive neighboring polymers are limited, the primary occurrence involves intramolecular reactions, leading to the formation of a percolation network with a lower fractal dimension than the prediction of the mean-field theories (~2.5) [80,81]. The fractal dimension depends on the concentration, leading to the variation in the *p*-dependence of *τ*_visco_.

## 5. Conclusions

Recent experimental studies on the molecular understanding of viscoelasticity in transient networks were reviewed. Transient networks exhibited the viscoelastic relaxation with a single mode. The relationships between the viscoelastic relaxation time and molecular dynamics were studied on the basis of the diffusion of component polymers and the binding kinetics of association points.

Key findings stated in this review can be summarized as follows: (i) the constitutive polymers diffuse approx. 100 times larger than its self-size during the viscoelastic relaxation time, regardless of network structure regularity, which cannot be explained by the conventional molecular models; (ii) the activation energy of viscoelastic relaxation agrees with that of the dissociation of association points, indicating that the dissociation is the elementary process of the relaxation; (iii) the viscoelastic relaxation time is longer and shorter, depending on the detailed network structures, indicating that the viscoelastic relaxation is determined by the time development of the survival rate of the “backbone”, the essential chains supporting the stress, not by the diffusion of polymers. The insights obtained from this review will provide foundational knowledge for the design of innovative transient network materials.

## Data Availability

The article is a review paper and no new data have been established yet.
